# Inclusive fitness is an indispensable approximation for understanding organismal design

**DOI:** 10.1111/evo.13739

**Published:** 2019-04-25

**Authors:** Samuel R. Levin, Alan Grafen

**Affiliations:** ^1^ Department of Zoology Oxford University South Parks Road Oxford OX1 3PS United Kingdom; ^2^ St John's College Oxford University Oxford OX1 3JP United Kingdom

**Keywords:** Fitness maximization, biological design, inclusive fitness, population genetics, δ‐weak selection, social evolution

## Abstract

For some decades most biologists interested in design have agreed that natural selection leads to organisms acting as if they are maximizing a quantity known as “inclusive fitness.” This maximization principle has been criticized on the (uncontested) grounds that other quantities, such as offspring number, predict gene frequency changes accurately in a wider range of mathematical models. Here, we adopt a resolution offered by Birch, who accepts the technical difficulties of establishing inclusive fitness maximization in a fully general model, while concluding that inclusive fitness is still useful as an organizing framework. We set out in more detail why inclusive fitness is such a practical and powerful framework, and provide verbal and conceptual arguments for why social biology would be more or less impossible without it. We aim to help mathematicians understand why social biologists are content to use inclusive fitness despite its theoretical weaknesses. Here, we also offer biologists practical advice for avoiding potential pitfalls.

Inclusive fitness was invented by Hamilton (1964) as an individual‐level quantity (page 8) that natural selection should cause organisms to act as if maximizing (page 17). The idea has been controversial for many decades (Cavalli‐Sforza and Feldman 1978) and there has been a recent explosion of controversy and debate (there are too many papers to cite here, but, e.g., see Nowak et al. 2010 and replies, e.g., Abbot et al. 2011; Bourke 2011; Queller 2016) . We endorse and adopt here the resolution offered by Birch (2017a,b), who accepts that the critics (e.g. Nowak et al. 2010; Allen and Nowak 2016) are right to point to technical difficulties in establishing that inclusive fitness is well defined *in a fully general theoretical model*, but at the same time concludes that the advocates (e.g., Grafen 2006; Abbot et al. 2011; Gardner et al. 2011; West and Gardner 2013; Marshall 2015, 2016; Queller 2016) have a strong enough case within certain assumptions (notably additivity of fitness effects) to adopt inclusive fitness as an organizing framework for understanding social behavior. A goal here is to set out in more detail why inclusive fitness is such a practical and powerful organizing framework, to such an extent that we argue the study of social behavior would become more or less impossible without it.

In the course of the recent debate, several authors (e.g., West and Gardner 2013; Queller 2016) have written very clear arguments for some of the advantages of inclusive fitness, and readers are encouraged to refer to these papers for a general discussion of the role of inclusive fitness in biology (West and Gardner 2013; Queller 2016). However, our admittedly narrower focus here is to address mathematically rooted criticisms of the assumptions required to guarantee inclusive fitness maximization, and the claim that measures such as mean‐offspring number do a better job at predicting gene‐frequency change. While this focus is narrower, it is also the controversial issue that continues to prevent productive dialogue between mathematicians and empiricists. Mathematical biologists making these points pay no regard to the practical arguments made by advocates of inclusive fitness, while still pointing to these formal shortcomings as a problem. Our goal here is to meet these mathematical arguments on their reasonable terms, and illustrate why, when interpreted in the light of whole‐organism biology, many of the problems fall away.

To achieve this, we first outline five advantages of inclusive fitness. We initially focus on these advantages under additivity, to make the points clearly in the absence of the offending complications. We then turn to to the problem of nonadditivity, and reconsider the advantages in this scenario. Finally, we discuss the importance of conditional behavior in the degree to which non‐additivity raises problems in practice, expanding on and clarifying points made previously (Grafen 1979; Queller 1996). We indicate how the necessary assumption of additivity can be checked in practical cases, and the likely impact of minor deviations. Levin and Grafen (Submitted) have shown formally that additive models are consistent with a very wide range of situations, and that inclusive fitness maximization does occur in model circumstances in which previous authors (Lehmann et al. 2015; Okasha and Martens 2016b) have failed to find it. Here, we focus on the less technical but broader conceptual arguments in support of those formal results, in a way that is accessible to nonmathematicians, and contains practical advice for empiricists.

## Inclusive Fitness under Additivity

Hamilton (1964) observed that adult offspring number, a standard metric of fitness, is affected not just by the actions of an individual but by those of the individuals it interacts with. Hamilton pointed out that measuring those effects of relatives involves averaging over possible distributions of genotypes, which in turn involves knowing gene frequencies in the population—a calculation he termed “unwieldy” (Hamilton 1964). However, he offered an alternative metric, which involves taking the perspective of the focal individual and its effects on others (as opposed to others' effects on it). He called this value “inclusive fitness,” and defined it as the sum of an individual's adult number of offspring in the absence of any social interactions (baseline fitness; more precisely, in the absence of social interactions in the performance of which there is genetic variability), and certain weighted effects the individual has on all individuals in the population, including itself. The effects are increases or decreases in offspring number caused by the individual, and the weightings are degrees of relatedness. Relatedness is a measure of genetic similarity between two individuals, with an individual having a relatedness of 1 to itself and 0 to a randomly selected member of the population. Inclusive fitness specifically does not include the effects of others on the focal individual.

Hamilton showed, under the assumption of weak selection, that this quantity, inclusive fitness, increases under selection, taking inspiration from Fisher's proof that standard fitness increases in an asocial model (Fisher 1930; Hamilton 1964, 1970), and modeling his technical argument on Kingman (1961). Hamilton argued that, as a result, we should expect organisms to appear as if they are trying to maximize their inclusive fitness (Hamilton 1970). For nearly 40 years, at least within behavioral and evolutionary ecology, most field and laboratory workers have treated inclusive fitness as the quantity that organisms appear designed to maximize, and tailored their studies and experiments accordingly (summarized in, e.g., Westneat and Fox 2010; Davies et al. 2012).

Inclusive fitness brings with it several advantages for the study of social behavior. Here, we outline five that we think are particularly important. In the following discussion, however, we are focusing on inclusive fitness under the assumption of additivity (although as we will see, all but the first extend beyond this restriction). There are two types of additivity. The first, “additive gene action,” is concerned with how different alleles combine within an individual to produce a phenotype, or social behavior. Considering two alleles, A and B, is the difference between being AA and AB the same as that between AB and BB (additivity), or different (nonadditivity)? The second type of additivity, which is of relevance here, refers to additivity of fitness effects between individuals. How does the effect of a social action combine with the existing number of offspring of an individual? And how do the effects of different social actions combine to affect one individual's offspring number? Let's say an individual has five offspring in the absence of social interactions, and social partners can choose to help that individual by giving it an extra “b” offspring. Does each instance of helping simply add the same number onto the individual's existing number of offspring (additivity), or do those fitness effects combine in some nonlinear way (nonadditivity). Simple inclusive fitness models, which are used to make predictions about animal behavior, assume additivity. We return to the problem of nonadditivity later, as it is central to Birch's (2017a, 2017b) resolution of the debate.

### ADVANTAGE 1: PREDICTING GENE FREQUENCY CHANGE

The first advantage of inclusive fitness is that, under additivity, it correctly predicts the direction of gene frequency change. Hamilton's rule provides a simple tool for doing so (Hamilton 1964). Given a trait that has an effect, in terms of adult offspring number, on its bearer, −c, and has an effect on social interactants, *b*, that trait will spread in the population if rb−c>0, where *r* is the relatedness to the recipients affected by the trait. More generally, genes whose bearers tend to have a higher value of inclusive fitness will be favored by natural selection (Hamilton 1964, 1970). The rule easily extends to multiple recipients, although it is crucial that there is just one actor. Note that we are referring to the simple form of Hamilton's rule derived by Hamilton (1964), in which the fitness effects are absolute effects on offspring number, as this form is sufficient under nonadditivity. We discuss the more general form (Queller 1992; Gardner et al. 2011) in Section 5.2.

However, this advantage is rarely important on its own. It is the connection with other properties that makes predicting gene frequency change important in practice. We now go on to articulate those other properties.

### ADVANTAGE 2: A DESIGN PRINCIPLE FOR INDIVIDUALS

Inclusive fitness provides a design principle for organisms. A fundamental question in biology (dating, in spirit if not detail, to Darwin) is how the dynamics of gene frequency change leads to the appearance of design and adaptation in organisms. Fisher's Fundamental Theorem (1930) provided such a link for non‐social traits, by proving that natural selection always tends to increase mean fitness. It sometimes then follows that organisms appear designed as if trying to maximize that quantity. Hamilton established a similar result to Fisher's, but for social traits. Inclusive fitness is a quantity that, under additivity, organisms should appear designed to maximize (Hamilton 1964; Queller 1992; Grafen 2006; Gardner et al. 2011; West and Gardner 2013; Lehmann and Rousset 2014; Rousset 2015; Lehmann et al. 2016; Taylor 2017).

Inclusive fitness is particularly useful as a design principle because it is can be conceptualized as an individual level property. Although it is possible to search for design principles at the level of the gene or the group, students of behavior tend to predict and measure organismal phenotypes. The selfish gene approach can be useful for certain gene level questions, such as intragenomic conflict (Haig 2002; Burt and Trivers 2006; Foster 2011; Gardner and Úbeda 2017), whereas group level principles have been less useful (West et al. 2007, 2008; Gardner and Grafen 2009; West and Gardner 2013). Individual level principles are the default tool of the trade (Davies et al. 2012), and have, in part, been successful because different loci in the genome tend to be selected in the same direction, and genetic rebels tend to be silenced by the “parliament of the genes” (Leigh 1977; Alexander and Borgia 1978; Strassmann and Queller 2010; West and Gardner 2013). As a result, the different tissues and organs within an individual work together for a common cause, the good of the majority group of genes, which for shorthand we often call the good of the organism (Leigh 1977; Haig 2002; Burt and Trivers 2006; Strassmann and Queller 2010; West and Gardner 2013).

If there is an individual level design principle in biology, then, at equilibrium, organisms should look like rational actors choosing among a suite of available phenotypes, the one that maximizes a certain quantity (Okasha and Martens 2016b). Hamilton showed that, within his assumptions, there was such a quantity—inclusive fitness.

### ADVANTAGE 3: INTERPRETING BEHAVIOR

Inclusive fitness provides a simple, economic interpretation of organismal behavior (Hamilton 1970; Grafen 1984; Frank 1998). Organisms should trade off their own offspring against those of another individual at a rate *r* (relatedness). This serves three purposes.

First, it helps generate testable predictions, even without complex mathematical models. Simple verbal reasoning can lead us to predict how many eggs a certain species of bird should lay each year, or how much food a cub should leave for its sibling, and these predictions are then readily testable.

Second, it guides us to new study systems by suggesting what biological features might lead to problematic or interesting cases. A heuristic for generating predictions is exactly how a scientific field makes progress, as has been demonstrated in the fields of behavioral ecology and evolutionary ecology (Krebs and Davies 1978, 1987; Charnov 1982; Krebs and Davies 2009; West 2009; Westneat and Fox 2010; Davies et al. 2012).

Third, it helps us understand social behavior by providing a way to reason about adaptations. For example, it is true that populations should be made up of genes that are associated with a higher contribution of gene copies to the next generation. But this does not tell us much about what kinds of traits and real life observations would defy our expectations, what population structures might lead to particularly unusual phenomena, or what adaptations (underpinned by many genes) might spread. Inclusive fitness offers us all of those things, by telling us that organisms should make decisions using this simple trade‐off in offspring.

### ADVANTAGE 4: EMPIRICAL TESTABILITY

An additional benefit of this simple trade‐off is that inclusive fitness predictions are testable in the laboratory and the field. Inclusive fitness, remarkably, does not require knowing the genetics of a trait (the ‘phenotypic gambit'), the genotypes of various individuals in the population, or even gene frequencies (Grafen 1984; West and Gardner 2013). We only need to know the fitness effects of the trait and the relatedness to the recipients. In practice, pedigree relatedness usually suffices (because it leads to the genes in the genome pulling in the same direction), making experiments surprisingly feasible (West and Gardner 2013). This is supported by the success of the vast body of empirical literature that has sprung from inclusive fitness theory (for an entry into that literature, see: Foster 2009; Davies et al. 2012), and for an attempt to quantify such successes (Abbot et al. 2011, Tables 1 and 2).

### ADVANTAGE 5: GENERAL APPLICABILITY AS TO THE EMPIRICIST

Hamilton (1964) made remarkably few assumptions (namely autosomal diploidy, outbreeding, semelparity, and weak selection). This means we can study populations in which there are many types of individuals with interactions occurring with any number of recipients. In general, our models, and therefore predictions, do not have to be custom fitted to each new species we study, especially useful considering we rarely know the genetic details to do so. This not only leads to more theoretically grounded empirical work, but provides for broad unification across the tree of life. An aim of any science is to have simple, overarching frameworks that work across specific details. In turn, this generality allows us to make and test comparative predictions, which hold across populations and species. Comparative statics are a bedrock of evolutionary biology, and the generality of Hamilton's theory lends itself to them (Darwin 1871; Parker and Maynard Smith 1990; Harvey et al. 1991; Harvey and Purvis 1991; Hughes et al. 2008; Cornwallis et al. 2010; Davies et al. 2012; Fisher et al. 2017; Cornwallis et al. 2017). For a further discussion of extensions to Hamilton's original paper, which have attempted overcoming some of the few assumptions he made, see, for example, Queller (1992), Grafen (2006), and Gardner et al. (2011).

## The Challenge of Nonadditivity

So far we have focused on inclusive fitness under additivity. Things get more complicated when we allow for fitness effects to combine nonadditively, a problem first dealt with formally in a general way by Queller (1985). Nonadditivity can arise a number of ways. For example, consider a simple two player game, in which players can either cooperate, giving their partner a fitness increment *b*, or defect. In an additive game, individuals receive *b* from cooperators regardless of their strategy. But in a nonadditive, synergistic game, when two cooperators interact, they get an additional (possibly negatively) synergistic effect, *d* (Queller 1985). This might occur, for example, if two hunters are more (or less) than twice as good as one. Nonadditivity arises when the fitness effects of social actions combine, either with the existing number of offspring of an individual or with each other, in a nonlinear manner. While effects on fecundity may often naturally be assumed to combine additively, effects on survival are more likely multiplicative.

### ADVANTAGE 1: PREDICTING GENE FREQUENCY CHANGE

The challenge nonadditivity poses for inclusive fitness has been discussed since at least 1978 (Cavalli‐Sforza and Feldman 1978; Uyenoyama and Feldman 1982; Karlin and Matessi 1983; Queller 1985). The problem is twofold. First, where before we needed to only know fitness effects and relatednesses to predict gene frequency change, we now need to know genetic makeup of the population, including the frequency of the gene (which will change under selection). Second, it is no longer even clear how to define inclusive fitness. Take, for example, the two player game described above. Inclusive fitness requires isolating the effects of the focal individual's genotype. But what portion of the synergistic component, *d*, is the focal individual responsible for?

Without a good way to define inclusive fitness in these scenarios, many authors turn to naive versions of inclusive fitness, such as “simple‐weighted sum,” which are definable under synergy. Simple‐weighted sum sums an actor's whole fitness and the fitness of all other individuals, weighted by their relatedness, which leads to double‐counting of fitness effects, and is, importantly, not inclusive fitness (Grafen 1982). A number of authors have shown that, for example, in a simple nonadditive two player game, such naïve versions of inclusive fitness wrongly predict the direction of gene frequency change (Grafen 1979; Queller 1985; Lehmann et al. 2015; Okasha and Martens 2016b; Taylor 2017).

Several authors (Grafen 1979; Lehmann et al. 2015; Okasha and Martens 2016b; Allen and Nowak 2016; Frank 2013) have also pointed out that using Hamilton's “neighbor‐modulated fitness” (NMF) resolves this problem in some scenarios (although these authors do not always acknowledge that they are dealing with NMF, instead referring to it by other names, such as “Grafen‐1979 payoff,” which is just NMF in a two player game). This is not surprising as NMF is simply mean number of adult offspring (it adds to the focal individual's fitness the offspring it would receive if its social partners expressed the same phenotype, weighted by the probability that they will, that is, the population frequency of altruism enhanced or diminished by relatedness). That it correctly predicts gene frequency change stems directly from the fact that offspring are how genes are passed on.

NMF's ability to make the right prediction under a wider range of circumstances has led several authors to suggest adopting offspring number (under its various guises) in place of inclusive fitness (Lehmann et al. 2015; Okasha and Martens 2016b). However, we proceed to discuss the ways in which mean offspring number is inferior to inclusive fitness with regards to the other advantages.

### ADVANTAGE 2: A DESIGN PRINCIPLE FOR INDIVIDUALS

First, offspring number is not a design principle. Hamilton's (1964) starting point was NMF because selection acts through offspring number. He developed inclusive fitness, despite it requiring more assumptions, because of its conceptual and practical advantages. In particular, inclusive fitness offers a design principle (advantage 2). It provides a link between gene frequency dynamics and design, because organisms can appear designed to maximize their inclusive fitness.

The same cannot be said for offspring number. As mentioned earlier, a design principle implies that organisms should appear to adjust their phenotypes to maximize a given quantity (Okasha and Martens 2016b). An organism cannot adjust its NMF, as her value of NMF is outside her control. NMF is determined by the genotypes, or identities of a focal individual's partners. Adjusting it would require adjusting partners’ genotypes. By analogy, offspring number is equivalent to “being‐part‐of‐a‐group‐of‐four‐ness” as a design principle (as contrasted with a simple propensity to join a group). Inclusive fitness, on the other hand, is under the control of the individual—an offspring simply has to adjust its own phenotype to alter its inclusive fitness (West and Gardner 2013). Hamilton (1964) showed that, at equilibrium, organisms should appear to be choosing traits with regards to inclusive fitness, and that this results from gene frequency change. Although critics have doubted that IF is under the individual's control in general, they do accept the principle under additivity (Lehmann et al. 2015; Okasha and Martens 2016b; Birch 2017a, 2017b). Thus, we lose the design principle if we use NMF, which sacrifices most of the utility of inclusive fitness.

### ADVANTAGE 3: INTERPRETING BEHAVIOR

Further, even if we were to stop using inclusive fitness for constructing models and designing experiments, its interpretive advantage (3) means that we would still use it to generate ideas, choose systems to study, and interpret social behavior, provided the effective trade‐off is still roughly given by relatedness. Inclusive fitness tells us that organisms should trade‐off others' offspring against their own at a rate *r*, for relatedness. These lead us to identify systems with relatedness asymmetries, large opportunities for helping or harming, unusual sex ratios, and extreme population structures as systems that would be fruitful for study. It also points us to traits that might disprove our theory. Traits that do not appear to abide by that valuation deserve further attention. With regard to such exceptions, we would like to know how far the trade‐off value is pushed away from *r*. We would also like to know whether different loci in the organism have their critical *r* pushed to the same extent or even in the same direction.

If the trade‐off value is not changed much, and changes inconsistently at different loci, then the complications will not alter the predictions of inclusive fitness very much. This is why it is important that no one who offers alternatives offers a useful interpretive principle, or explains how far the existing principle is really compromised.

### ADVANTAGE 4: EMPIRICAL TESTABILITY

Even if we stopped using inclusive fitness to construct models, we would struggle to continue our empirical work. The reason is straightforward: when you stop using inclusive fitness, you start needing to know genetics (Grafen 1984). To test a prediction from inclusive fitness theory, we must observe which individuals act and calculate rb−c for those actors. We might use information on who acts (and who does not) to estimate *b* and *c*, by subtracting average offspring number of non‐actors from actors, and of non‐recipients from recipients. More generally, we can regress the average adult offspring number on (i) the number of actions taken and (ii) the number of actions received. Thus even without knowing genotypes, we can apply inclusive fitness.

For NMF the situation is more complicated, and we need to know genotypes. In the very simplest haploid two‐allele model, higher average NMF of an allele, H, compared to an alternative allele, N, tells us that H will be selected. But if strategies include rare deviant behavior, and therefore the opportunity to act occurs only with some small probability δ<<1, then only knowing NMF and who acts is not sufficient; instead, genotypes are needed. This is because an actor will rarely be a recipient, and so the actors do worse, even though the trait may be favoured by acting on relatives. On the basis of phenotypes, those relatives are counted in among the non‐actors, raising the NMF of non‐actors (see more detailed discussion of rare deviant behavior in Section 4). We would need to know genotypes to add them to the actors, and to show that possessing the tendency to act was beneficial.

To recover a maximisation principle in the field, then, we need genotypes. Then we can obtain maximisation of NMF by averaging over the NMF of H allele‐bearers and comparing that with N‐allele bearers. At this point, one familiar with modelling may be confused, as models of NMF include relatednesses, and a mathematically equivalent NMF version of Hamilton's rule can be extracted. However, in the field, relatednesses are not needed for NMF – a simple count of mean offspring number already includes this information. However, knowing who to count requires knowing genotypes. Specifically, to average over bearers of the allele in question, we need to know the genotypes of the individuals we study (usually impractical) and the genetics of the trait in question (which we rarely do in practice). This “epistemological argument” was made briefly by Grafen (1984), and we have attempted to expand on it here.

On the other hand, inclusive fitness offers the biologist a measure of phenotype that predicts evolutionary change. Fortunately, the phenotypic gambit, of assuming we do not need to know the genetic architecture of a trait, has proved remarkably successful (Grafen 1984; West and Gardner 2013; Davies et al. 2012).

It is also worth noting that the NMF approach does not involve identifying the fitness consequences of a social action. Rather, we need to know only the genotype and the number of offspring. Indeed, one would conclude that a gene was spreading or not, and not know whether the cause was social behavior or pathogen resistance or liver‐enzyme activity. To study social behavior, we *should* investigate how the actions of one individual affect the number of offspring of another – that is what social behavior is. Alternatives to inclusive fitness, such as NMF, do not offer the empirical utility of inclusive fitness.

### ADVANTAGE 5: GENERAL APPLICABILITY AS TO THE EMPIRICIST

Inclusive fitness offers practical advantages to the modeler. Some authors (Nowak et al. 2010; Allen et al. 2013; Allen 2015; Allen and Nowak 2015; Nowak and Allen 2015; Akçay and Van Cleve 2016; van Veelen et al. 2017) have suggested abandoning inclusive fitness for what they refer to as “standard natural selection” models, which track gene frequencies. This approach is good at predicting gene frequency change in mathematical models. However, it requires generating a custom model for each new biological scenario. Inclusive fitness is a single framework that works across systems, independent of many (though of course not all) details. Hamilton's original model is surprisingly general, allowing both the theoretician and the empiricist to apply the ideas to systems with arbitrary numbers of interactions and many different kinds of individuals. This degree of generality and unity is a rare and sought after gift in the sciences.

Of course, there will always be limitations to validity, and the more these are understood the better. Recent critiques of inclusive fitness (e.g., Nowak et al. 2010; Allen and Nowak 2015) might possibly be put to good use in that direction, although few new issues of significance have been brought to light since 1978 (Cavalli‐Sforza and Feldman 1978).

## Conditionality

Before we proceed to discussing practicalities for behavioral ecologists, a simple model will help illustrate some of the above points. It is often pointed out that NMF and inclusive fitness calculations are mathematically equivalent, but what is less often clearly articulated is how they become distinct in practice. Here, a model makes the distinction clear, and shows how conditional behavior brings to light the difficulties of applying NMF.

All behavior is conditional, and models incorporating conditionality are important for understanding one of the advantages of inclusive fitness. In the unconditional case usually studied of inclusive fitness in a grouped population, a standard infinite haploid model with groups of size *n* is first introduced, with *p* as the frequency of an altruism allele, *A*, and using *r* for relatedness, in the simplest way we write the average number of other altruists in the group of a randomly chosen altruist as $n_{A}=\left(n-1\right)\left(r+\left(1-r\right)p\right)$, and the average number of altruists in the group of a randomly chosen non‐altruist (*B*) as $n_{B}=\left(n-1\right)\left(1-r\right)p$. We assume an altruist suffers a cost of *c* and gives *b* to each other group member.

Hamilton (1964) identified two measures of fitness for predicting gene frequency change. NMF is simply a measure of mean offspring number, which sums an individual's fitness in the absence of social interactions and the effects of all individuals in the population on that individual. Inclusive fitness (IF) sums baseline asocial fitness, and the effect the actor has on all individuals, including itself, weighted by relatedness. The mean IF and mean NMF of A and B in this model are

$$\begin{array}{ccc} \text{NMF} & = & \left\{\begin{array}{cc} 1-c+n_{A}b & \text{altruist}\\ 1+n_{B}b & \text{non-altruist} \end{array}\right.\\ \text{IF} & = & \left\{\begin{array}{cc} 1-c+(n-1)rb & \text{altruist}\\ 1 & \text{non-altruist} \end{array}\right.. \end{array}$$

When we substitute as indicated for $n_{A}$ and $n_{B}$ we obtain

$$\begin{array}{ccc} \text{NMF} & = & \left\{\begin{array}{cc} 1-c+(n-1)(r+(1-r)p)b & \text{altruist}\\ 1+(n-1)(1-r)pb & \text{non-altruist} \end{array}\right.\\ \text{IF} & = & \left\{\begin{array}{cc} 1-c+(n-1)rb & \text{altruist}\\ 1 & \text{non-altruist} \end{array}\right., \end{array}$$

and find that the mean differences for altruist minus non‐altruist, which predict the spread of altruism, are

$$\begin{array}{cc} \text{DNMF} & =-c+(n-1)rb\\ \text{DIF} & =-c+(n-1)rb. \end{array}$$

Thus, NMF and IF predict the spread of the altruism allele in exactly the same cases. However, note even in this simple case that NMF and IF differ: in particular, NMF includes the altruism provided by the background fraction of altruists ((n−1)(1−r)pb) for both altruists and non‐altruists. The sum of these terms is the diluting factor of Hamilton (1964), and its presence in a model is a sign that NMF rather than IF is used. For example, the important work of Rousset (2004) on the evolution of social behavior in structured populations employs NMF. In recent work, in which altruists are always rare so that P=0, the difference technically between NMF and IF can be hard to make (e.g., Lehmann et al. 2015).

However, in a conditional model, the difference remains very clear when altruists are rare. Now, we amend our model so that in each group one individual is selected at random to be the potential altruist, and a random other individual is selected to be the potential recipient. The probability that the actor will be an altruist will be $P_{A}=1/n+\left(\left(n-1\right)/n\right)\left(r+\left(1-r\right)p\right)$ for the group to which a randomly selected altruist belongs and $P_{B}=\left(\left(n-1\right)/n\right)\left(1-r\right)p$ for the group of a randomly selected non‐altruist. We distinguish by suffices on fitnesses (NMF and IF) between the fitnesses of (i) potential altruists (suffix PA), (ii) potential recipients (PR), and (iii) unselected individuals (US). The NMF and IF are now

$$\begin{array}{ccc} \text{NMFUS} & = & 1\\ \text{IFUS} & = & 1\\ \text{NMFPR} & = & \left\{\begin{array}{cc} 1+p_{A}b & \text{altruist}\\ 1+p_{B}b & \text{non-altruist} \end{array}\right.\\ \text{IFPR} & = & 1\\ \text{NMFPA} & = & \left\{\begin{array}{cc} 1-c & \text{altruist}\\ 1 & \text{non-altruist} \end{array}\right.\\ \text{IFPA} & = & \left\{\begin{array}{cc} 1-c+rb & \text{altruist}\\ 1 & \text{non-altruist} \end{array}\right.. \end{array}$$

Substituting as indicated for $P_{A}$ and $P_{B}$, we find that the mean differences for altruist minus non‐altruist are

$$\begin{array}{ccc} \text{DNMFUS} & = & 0\;\text{DNMFPR}=rb\left(\frac{n-1}{n}\right)+\frac{b}{n}\;\text{DNMFPA}=-c\\ \text{DIFUS} & = & 0\;\text{DIFPR}=0\;\text{DIFPA}=-c+rb. \end{array}$$

The obvious interpretations are that an altruist always reduces its NMF by its action, while IF predicts that an altruist will spread if rb−c>0. One interesting question is which of these quantities is likely to be observable in the absence of genetic information, if all we can observe are the actions, and the offspring numbers of the individuals. We assume that by direct sequencing or pedigree information or demographic modeling we can estimate *r*. By observing actual actors (AA) and actual recipients (AR), compared to uninvolved individuals, we can estimate the mean offspring number of US, AA, and AR. This yields an estimate of *b* (AR−US) and *c* (AA−US), from which we can calculate inclusive fitnesses. However, owing to ignorance of *p*, we cannot estimate most of the NMF values.

A second point is that it is not true that selection favors altruism if the NMF of realized altruists is greater than the average NMF. We would need to average in the NMF of actual recipients, but we cannot distinguish the genetic altruists from the genetic non‐altruists, so we do not know which recipients to include in that average. Thus the correct mathematical statement that NMF predicts gene frequency changes applies in the theoretical situation that we know the genotypes of all the individuals, but not in the common empirical situation where we can observe only the actions.

In a more realistic situation, in which altruism opportunities arise randomly across the groups, and in which the chance of taking up an opportunity is genetically multifactorial, the simplicity of the inclusive fitness approach remains, while the NMF approach becomes more and more enmired. The theoretical and usual empirical situations are thus very distinct, and these differences need to be respected.

This simple model illustrates that even if NMF works better in a wider range of theoretical scenarios, as has been pointed out for decades (Hamilton 1964; Grafen 1979; Lehmann et al. 2015) it may not be a useful practical tool. We now turn to the question of what behavioral ecologists can make of these challenges.

## Practicalities for Behavioral Ecologists

The previous discussion suggests that, while offspring number is useful for predicting gene frequency change in mathematical models, for those interested in social behavior and design, it is not a viable option. Offspring number, being outside the control of the individual, cannot be an individual level design principle. Further, measuring predictions using NMF usually requires knowing the relationship between genotype and phenotype, and being able to measure genotypes, something that is for now impractical in the field (and usually the laboratory, too). How should whole organism biologists proceed, then, if they were to aim to work without using the concept of inclusive fitness? We see three options.

### ABANDON DESIGN

The limitations of inclusive fitness have led some authors to call for abandoning an individual level design principle altogether (Nowak et al. 2010; Doebeli 2010; Allen et al. 2013; Allen 2015; Allen and Nowak 2015; Nowak and Allen 2015; van Veelen et al. 2017). However, none of these authors provide (i) an alternative explanation for design, (ii) a consistent, unified way to generate predictions, or (iii) an adaptive principle that can be tested in the field and the laboratory. Instead, they offer no design principle, and suggest making custom models for each new situation, usually using metrics that will be impractical to measure empirically, such as genotypes and the relationship between genotype and phenotype. It is therefore unsurprising that inclusive fitness has a huge empirical literature and the alternatives essentially none (Krebs and Davies 1978, 1987; Charnov 1982; Krebs and Davies 2009; West 2009; Westneat and Fox 2010; Davies et al. 2012). This strengthens Birch's resolution that inclusive fitness offers a useful organizing framework, and goes further in highlighting its practical and empirical utility (Birch 2017a, 2017b).

While the alternative approaches are useful for theoretical models of gene frequency change, when it comes to social behavior, we see exquisite design, which demands explanation. Further, theories must make predictions that can be tested on real organisms. To be clear, we mean that hypotheses about social behavior are tested using the working hypothesis that inclusive fitness is maximized: we do not mean that it is usually possible to test whether inclusive fitness is in fact maximized. That would require the same kind of genetic information that we argue is currently vanishingly rare and likely to remain rare. For students of social behavior, abandoning the design approach is not a viable option. Fortunately, the design approach has been spectacularly successful. A more detailed discussion of the utility of adaptationism can be found elsewhere (Welch 2017; Gardner 2017).

### A NONADDITIVE HAMILTON'S RULE

Another option is to rewrite Hamilton's rule so that it makes correct predictions. Hamilton's rule is an inclusive fitness tool used for predicting the direction of selection. As we have said, inclusive fitness is undefined to the extent that fitness effects are strictly nonadditive. Some authors have pointed out that one option is to redefine components of Hamilton's rule to make it fully general, even allowing for nonadditive interactions (Queller 1985, 1992; Gardner et al. 2011; Queller 2011; Rousset 2015; Taylor 2016; Lehmann et al. 2016; Taylor 2017). In the standard approach, *b* and *c* are effects on offspring number, and *r* is a measure of genetic similarity between two individuals. If we replace these values with regressions on fitness, we recover a fully general Hamilton's rule, which does not require additivity and always correctly predicts the direction of evolutionary change (Queller 1992; Gardner et al. 2011; Rousset 2015). Depending on the causal breakdown we desire, nonadditive effects can be incorporated into their own term (Queller 1992, 2011), or, alternatively, we can leave the fitness effects (*b* and *c*) unchanged, but replace *r* with a higher order relatedness coefficient, for example one that captures the relatedness between a focal individual and a pair of recipients (Taylor 2016, 2017). Both of these are very valuable theoretical advances, showing the complete generality of Hamilton's rule when parameters are chosen correctly.

However, as various authors have pointed out (Birch and Okasha 2014; Birch 2014; Taylor 2016, 2017; Allen and Nowak 2016; Okasha and Martens 2016a), the cost of this generality is a loss in simple interpretation of the terms. They can no longer be understood as simple effects on offspring number, we no longer have a simple interpretation of social behavior, and, without knowing genetics, the parameters are no longer easily measurable in the field and in the laboratory. Recently some authors (Nowak et al. 2017) have confused this general, regression form of Hamilton's rule with the simple one discussed here. While it's true that this general form of Hamilton's rule (sometimes referred to as ‘HRG’, Birch 2014) gains generality at the cost of empirical utility, the simple Hamilton's rule we have discussed (or versions of it), defined in terms of effects on offspring number, is the one that has been used to enormous empirical success, as outlined above and reviewed by, for example, Foster (2009), Abbot et al. (2011) (Tables 1 and 2), Bourke (2011), and Davies et al. (2012). Indeed, critics of the general form of Hamilton's rule have not offered an alternative that rivals the empirical utility of standard Hamilton's rule. The regression approach is a powerful conceptual advance (Rousset 2015), but not empirically useful in the usual situation that the genetics of the individuals studied are unknown.

### USING ADDITIVE INCLUSIVE FITNESS AS AN APPROXIMATION

A final option, then, is to use additive inclusive fitness as an approximation, and remain alert to when this approximation will fail (and by how much). Grafen (1985) has a list of reasons why additivity is probably nonproblematic in practice. We discuss one example here, explaining how mutations of rare but possibly large effect (similar to the population genetic notion of “penetrance”) can resolve the usual problems that arise from nonadditivity. This resolution has been discussed by a number of authors in specific cases, and here we argue for it being a potentially general solution (Queller 1996; Grafen 1979; Birch 2017a).

Nonadditivity creates a problem for inclusive fitness in that fitness effects (and therefore, changes in gene frequency) are no longer wholly attributable to a focal genotype. For example, consider a simple two player game with discrete strategies, where each player can choose to play either Cooperate (to give *b* at cost *c*) or Defect, and where when two cooperators interact they receive an added effect, *d*. A cooperator will have many occasions on which she encounters another cooperator, and how likely these occasions are depends on the degree of relatedness, or assortation, in the population (*r*). If we imagine a mutant in the population that played Cooperate instead of Defect, increasing *r* increases the likelihood that its partner's strategy will also be Cooperate, and inclusive fitness fails to take this alteration in the partner's behavior into account. As a result, a naïve version of inclusive fitness makes the wrong prediction in a discrete, nonadditive, two‐player game (Grafen 1979; Okasha and Martens 2016b).

However, if strategies are not discrete but continuous, where a player can choose to cooperate a fraction π of occasions, the situation changes. Now, a variant strategy plays Cooperate π+δ portion of occasions. In other words, it plays Cooperate instead of Defect on one occasion out of many, and the probability that it is the same occasion its related partner also plays Cooperate instead of Defect (because of the mutant strategy—it may often play Cooperate in absolute terms) is very low (Grafen 1979).

This principle extends beyond simple two‐player games. More generally, when the genetic component of the variability in how individuals act on any given occasion is proportionally low (which implies the δ‐weak selection of Wild and Traulsen 2007), we can use inclusive fitness to make accurate predictions. In this case, the only way *r* impacts the direction of selection is through an actor's vested interest in its social partners. When this type of variability is high, *r* also determines assortation of strategies, which inclusive fitness does not capture. Fortunately, a low genetic component of variability will be the norm for populations near equilibria, where it is usually reasonable to suppose we study organisms (Fisher 1930; Grafen 1985), a point endorsed by Birch (2017a, 2017b). Thus, for traits of interest to behavioral ecologists, inclusive fitness should often make the correct predictions even under nonadditivity.

The mathematical importance of δ‐weak selection has been discussed elsewhere (e.g., Taylor and Frank 1996; Wild and Traulsen 2007; Peña et al. 2015; Levin and Grafen, Submitted). Our point here is to explain the kinds of biological scenarios that deliver this mathematical convenience, extending brief verbal arguments by Grafen (1979) and Queller (1996). In a companion paper (Levin and Grafen, Submitted), we formalize this otherwise verbal argument and discuss two recent papers that look for inclusive fitness maximization but fail to find it (Lehmann et al. 2015; Okasha and Martens 2016b), both coming to the conclusion that expected offspring number (‘$u_{B}$’ in Lehmann et al. 2015 and “Grafen 1979” in Okasha and Martens 2016b) is a better measure. Levin and Grafen (Submitted) show that probabilistic mixing of phenotypes recovers inclusive fitness maximization.

We also note that this type of probablistic mixing may also resolve some questions about how inclusive fitness moves from the level of the trait to the individual. Queller (1996) has argued that certain types of nonadditivity can make defining inclusive fitness at the individual‐level difficult, because different measures are required for different traits. Specifically, when individuals adopt different roles in an interaction, it is not always clear how to assign offspring number to the control of one actor, analogous to the challenges of assigning offspring number when there is synergy between traits. In the absence of a formal analysis, we suspect that this type of nonadditivity will also be resolved by allowing probabilistic mixing. In the meantime, we are reassured that Grafen (2006) allowed different types of social actions, including unique roles, and still recovered inclusive fitness maximization.

Finally, we have assumed that intra‐organismal conflicts (e.g., genomic imprinting) are not pulling organisms away from inclusive fitness optima. The effect of conflict on inclusive fitness equilibria is interesting, but beyond the scope of this paper (for an entry into that literature, see, e.g., Haig 2002; Foster 2011; Gardner and Úbeda 2017). Genetic conflict would indeed very likely require genetic genetic knowledge to investigate.

### MONITORING ASSUMPTIONS

Of course, effective nonadditivity may not always hold. Fortunately, theory tells us what to be on the lookout for. For example, recent environmental change may mean populations are not near equilibria, and therefore additive genetic variability may be high. This is a caveat that applies to all evolutionary biologists, not just those studying social dilemmas. More specifically, we might suggest that students of social behavior be on the lookout for clear assortation of actions in nature.

As we have said, nonadditivity is problematic when there is strong assortation of actions, because inclusive fitness calculations do not take that additional effect of relatedness into account. This is something a field or laboratory worker can observe. For example, consider a population of birds in a wood. If relatives are not interacting, we would not expect *strategies* to be correlated. However, if relatives do interact, but the genetic component of the variability in how individuals act on any given occasion is proportionally low (the δ‐weak selection of Wild and Traulsen 2007) we still would not expect *actions* to correlate between interacting individuals. The reason, as stated above, is that the chances of two interacting relatives expressing the deviant action on the same occasion are low. To be clear, we use the phrase “on the same occasion” to illustrate the point, but technically it does not refer to a set of different occasions that always arise (in which case nonadditivity can arise even when only one individual possesses the trait), but rather to different possible occasions, only one of which arises (when nonadditivity weakens because the chance of both being deviant is a lower order probability).

If we do observe clear assortation of deviant actions between partners in nature, it can be taken as a red flag that individuals may be engaged in a discrete game, and in this case, inclusive fitness may give the wrong answer (*if* the payoffs are also strongly nonadditive). This kind of discreteness might be most likely to arise in bacteria, because they are more likely to have single gene phenotypes. It may turn out that situations that generate problems for inclusive fitness are rare in nature. Either way, they do not require abandoning inclusive fitness. Instead, they serve as specific caveats for which to be on the lookout when conducting experiments.

It is worth considering one more aspect of the failure of inclusive fitness. Take, for example, situations in which inclusive fitness would not hold, due to high additive genetic variability and strongly nonadditive fitness effects. Are these exceptional cases consistent, in the sense that they make some consistent prediction as to how we should expect organisms to look or behave? Should they be more social than inclusive fitness predicts? Should they value the effects of their actions on others at r+ some predictable σ? Queller (1985) has suggested that in some cases, including simple two player games, the sign of the nonadditive component, *d*, contains some information about the direction selection will proceed in.

More generally, two questions are relevant to empirical biologists exploring this issue. Is there some design principle other than inclusive fitness, or is inclusive fitness the central target, with exceptional cases unpredictably moving organisms off the mark in varying directions? And if there is some other central target, does it differ from inclusive fitness in a way we could reliably measure? We surmise, in the absence of relevant work, that deviations depend on details of the genetics in an unilluminating way (unless one happens to know the genetics), although of course we would be very interested in any theoretical argument that claims to show the contrary.

## Conclusion

If we are interested in exact predictions of gene frequency change in mathematical models, offspring number is the measure of fitness we should use. However, if we are interested in social behavior and design, and in particular behavior and design in nature, we should use inclusive fitness under approximate additivity. It does have some limitations. But the alternatives are worse. And despite its limitations, inclusive fitness has many great conceptual and practical advantages for biologists. Further, as we have argued here and illustrated elsewhere (Levin and Grafen, Submitted), some of the theoretical limitations may disappear under biologically realistic scenarios.

If inclusive fitness is applicable, then all biological principles of social behavior are equivalent to it. If inclusive fitness is not applicable, then we need to know genetics, and therefore, there can be no biological principle of social behavior. Thus, the significant questions are: how good an approximation is the inclusive fitness approach, and does it allow the subject of social biology to exist? For the moment, it is consistent with what little we know that the approximation is reasonable, and the empirical successes of social biology back up this conclusion.

Thus, the continuation of work with inclusive fitness is founded on a sophisticated notion of what assumptions are required for exactness of inclusive fitness, the consequences of likely deviations, and the assurance from empirical successes that the working hypothesis is by and large satisfactory. The cost of the nuance of this notion is that it is not *easily* captured in a fully general model. But it is conceptually more suited to the various roles inclusive fitness plays within biology than the mathematically general models of population geneticists. Not only is inclusive fitness a powerful organizing framework (Birch 2017a, 2017b), but without it, we would have no useful theoretical approach for understanding social behavior in the laboratory, in the field, and in comparative work.

Associate Editor: L.‐M. Chevin

Handling Editor: M. Servedio
